# Antibiotic treatment can exacerbate biofilm-associated infection by promoting quorum cheater development

**DOI:** 10.1038/s41522-023-00394-4

**Published:** 2023-05-18

**Authors:** Lei He, Huiying Lv, Yanan Wang, Feng Jiang, Qian Liu, Feiyang Zhang, Hua Wang, Hao Shen, Michael Otto, Min Li

**Affiliations:** 1 Department of Laboratory Medicine, Ren Ji Hospital, Shanghai Jiao Tong University School of Medicine, 160 Pujian Road, Shanghai, 200127 China; 2 Department of Orthopedics, Shanghai Jiao Tong University Affiliated Sixth People’s Hospital, 600 Yishan Road, Shanghai, 200233 China; 3 Pathogen Molecular Genetics Section, Laboratory of Bacteriology, National Institute of Allergy and Infectious Diseases, U.S. National Institutes of Health, 50 South Drive, Bethesda, MD 20814 USA; 4 Faculty of Medical Laboratory Science, Shanghai Jiao Tong University School of Medicine, 227 South Chongqing Road, Shanghai, 200025 China

**Keywords:** Antimicrobials, Biofilms, Pathogens

## Abstract

Quorum cheating, a socio-microbiological process that is based on mutations in cell density-sensing (quorum-sensing) systems, has emerged as an important contributor to biofilm-associated infection in the leading human pathogen *Staphylococcus aureus*. This is because inactivation of the staphylococcal Agr quorum-sensing system leads to pronounced biofilm formation, increasing resistance to antibiotics and immune defense mechanisms. Since biofilm infections in the clinic usually progress under antibiotic treatment, we here investigated whether such treatment promotes biofilm infection via the promotion of quorum cheating. Quorum cheater development was stimulated by several antibiotics used in the treatment of staphylococcal biofilm infections more strongly in biofilm than in the planktonic mode of growth. Sub-inhibitory concentrations of levofloxacin and vancomycin were investigated for their impact on biofilm-associated (subcutaneous catheter-associated and prosthetic joint-associated infection), where in contrast to a non-biofilm-associated subcutaneous skin infection model, a significant increase of the bacterial load and development of *agr* mutants was observed. Our results directly demonstrate the development of Agr dysfunctionality in animal biofilm-associated infection models and reveal that inappropriate antibiotic treatment can be counterproductive for such infections as it promotes quorum cheating and the associated development of biofilms.

## Introduction

Healthcare-associated (nosocomial) infections (HAIs) affect an average of 7.6% of hospitalized patients in high-income countries^1^. In the United States, the mortality due to HAIs has been estimated at ~99,000 annual deaths^2^ and remains substantial despite some recent decline^3^. In low-income countries, HAI incidence and mortality are even higher^1,4^. Many HAIs stem from infections of indwelling medical devices, such as surgical implants, prosthetic devices, and urinary tract, intravenous, or central line-associated catheters^5^. The frequently fatal outcome of device-associated infections is mostly due to bloodstream infections that originate from the infected devices^6,7^. For example, central line-associated bloodstream infections (CLABSIs), one of the most deadly types of HAIs, have a mortality rate of 12–25%^8^. Device-associated infections are notoriously difficult to treat due to the formation of biofilms. Biofilms are bacterial agglomerations that can cover the device’s interior and exterior surface and exhibit pronounced resistance to antibiotics^9–12^. For that reason, device-associated infections have remained particularly difficult to treat even by modern drug development strategies^13^.

One of the most frequent causes of device-associated infections is *Staphylococcus aureus*^14^. In comparison to other bacteria often found in these infections, such as the closely related coagulase-negative staphylococci, device-associated infections with *S. aureus* typically show more severe clinical progression and outcome due to the more pronounced virulence of *S. aureus*^14–16^. *S. aureus* is infamous for its exceptional recalcitrance towards antibiotic treatment^17^, which is in part due to specific antibiotic resistance genes, such as *mecA* in methicillin-resistant *S. aureus* (MRSA)^17,18^. However, even antibiotics that normally efficiently kill MRSA, such as vancomycin, have strongly reduced activity against MRSA in biofilms^19,20^, and biofilms formed by methicillin-sensitive *S. aureus* (MSSA) also show pronounced resistance to antibiotic treatment^21,22^. Therefore, understanding the mechanisms of antibiotic resistance in biofilm infection is essential to improve therapeutic strategies for this major nosocomial pathogen.

Over the last decades, there has been intensive research into the molecular underpinnings of biofilm formation. In *S. aureus* and other biofilm-forming pathogens, this has led to the identification of a wide variety of molecules that in a structural, metabolic, or regulatory capacity, participate in biofilm formation^23,24^. For example, quorum-sensing (QS) systems, which adapt bacterial physiology to the changing conditions brought upon by increasing cell density, have a key role in biofilm development in many bacteria^25,26^. In *S. aureus*, there is one QS system, which is called Agr for accessory gene regulator^27,28^. Agr controls several factors that contribute to biofilm development, among them proteases and nucleases that degrade the biofilm matrix and phenol-soluble modulins (PSMs), which are amphipathic peptides that structure biofilms by preventing non-covalent interactions between matrix macromolecules^23,29,30^. The absence of PSMs leads to decreased formation of biofilm channels and overall thicker and more compact biofilms, to a similarly pronounced degree as seen in *agr* mutants, indicating a key role of PSMs in biofilm development^31^. We also confirmed the role of Agr and PSMs in *S. aureus* and *S. epidermidis* in models of biofilm-associated infections^25,31–34^. However, these represent rather rare examples in which biofilm infection models were used to confirm the validity of in vitro findings^25^. Generally, the dynamics of biofilm infection remain incompletely understood.

We previously reported that Agr-dysfunctional *S. aureus* mutants develop in vitro to a considerable extent^32^. The development of QS-dysfunctional mutants such as *S. aureus* Agr-dysfunctional mutants are commonly called “quorum cheating” and was first observed for the QS systems of *Pseudomonas aeruginosa*^35^. Quorum cheating describes the socio-microbiological phenomenon that some bacteria in a population shut off the costly, QS-controlled secretion of degradative enzymes that are needed for nutrient acquisition, relying on other members of the population until a balanced equilibrium of cheaters and non-cheaters develops^36^.

While established predominantly using in vitro research, there is evidence that similar principles of quorum cheating and quorum cheating control also apply to in vivo infection^37,38^. Importantly, we and others have reported that Agr-dysfunctional mutants can be frequently found in *S. aureus* infection isolates and particularly in those isolated from biofilm-associated infections or blood infections, which often stem from biofilms on infected indwelling devices^39–43^. Recent animal research in our laboratory has indicated that the development of *agr* mutants in biofilm-associated infections does not entirely follow predictions from socio-microbiological models^32^ inasmuch as it is strongly dependent on the specific in vivo setting, with the increased biofilm formation of *agr* mutants mediating increased resistance to innate immunity mechanisms. In accordance with this apparent key importance of Agr dysfunctionality for immune evasion in a biofilm setting, we also recently found, using analysis of sequential isolates, that Agr-dysfunctional mutants develop during human clinical PJI^40^, an observation made afterward in similar form in cystic fibrosis^44^. Notably, in our previous study, clinical biofilms were virtually 100% composed of *agr* mutants (“quorum cheaters”) rather than of an equilibrium of Agr-functional and -dysfunctional bacteria as the socio-microbiological model would have predicted^32^. The fact that in this recent clinical study, patients were commonly under antibiotic therapy prompted us to investigate in the present study, using animal models of biofilm infection, whether antibiotic therapy plays a role in the development of antibiotic-resistant biofilms via a possible stimulation of quorum-cheating effects.

## Results

### Quorum cheater development in biofilm and planktonic settings in vitro

**Fig. 1:**
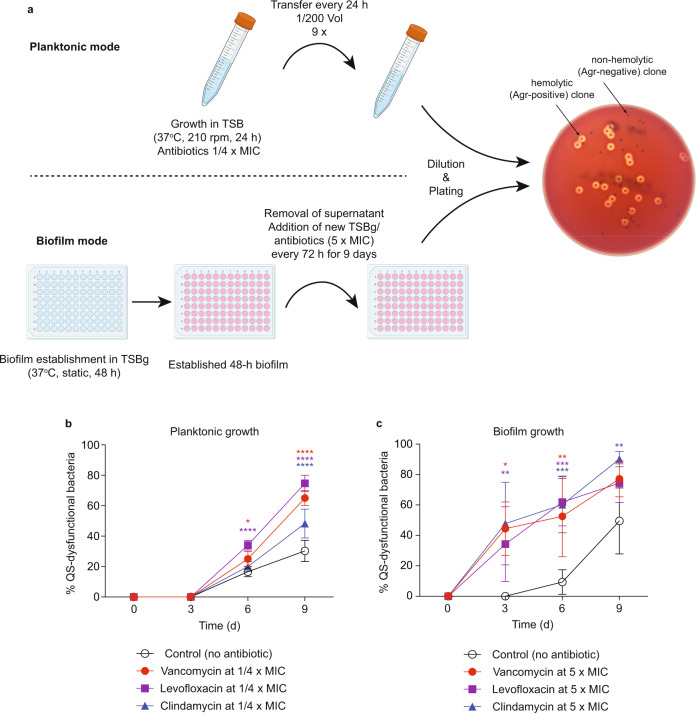
Development of Agr (QS)-dysfunctional mutants under antibiotic treatment in vitro. **a** Experimental setup. For planktonic growth, cultures of *S. aureus* LAC were inoculated daily into new TSB media at 1/200 volume for 9 days. Levofloxacin (Lev), vancomycin (Van), or clindamycin (Cli) were supplied at ¼× MIC. For biofilm growth, cultures of *S. aureus* LAC were inoculated at 1/200 volume from pre-cultures into microtiter plate wells containing 200 μl of TSBg each and grown for 48 h to form a biofilm. Then, the supernatant was gently removed, and 200 μl TSBg containing Lev, Van, or Cli was dispensed into the wells at 5× MIC In the following 9 days, every 3 days, the old supernatant was gently removed and replaced with fresh TSBg (200 μl) with the respective antibiotics. **b**, **c** Results for planktonic (**b**) and biofilm (**c**) growth. Agr dysfunctionality was assessed by hemolysis on sheep agar plates. *n* = 3/group and time point. Error bars show the mean ± SD. Statistical analysis is done by two-way ANOVAs with Dunnett’s post-tests versus control values.

We first analyzed the development of Agr-dysfunctional mutants under treatment with subinhibitory concentrations of antibiotics in vitro, using the common approach to determine hemolytic capacity as a proxy for Agr dysfunctionality^32,39,41,45^ (Fig. 1a). We used ¼× MIC concentrations for the planktonic and 5× (planktonic) MICs for the biofilm mode of growth^40^. These concentrations were chosen as they produced similarly modest growth inhibition under the conditions of the experimental setup (Supplementary Fig. 1). Vancomycin (Van), levofloxacin (Lev), and clindamycin (Cli) were chosen as antibiotics for this experiment because these were most frequently used for PJI in our clinic and because we also had used them in our previous study on antibiotic tolerance mediated by Agr-dysfunctional biofilms^40^. We used the MRSA strain LAC (USA300) for our experiments, which is nowadays a widely used standard strain in *S. aureus* pathogenesis research. USA300 isolates are the most common cause of community-associated MRSA and a leading cause of hospital-associated MRSA infections in the United States^46^.

In this experiment, we passaged cultures by daily inoculation of fresh growth media with 1/200 volume of the cultures in the planktonic mode setup. For the biofilm setup, as biofilms cannot be quantitatively transferred, we exchanged the supernatant on top of the biofilms grown in microtiter plate wells every three days with fresh growth medium. We found that the applied subinhibitory concentrations of antibiotics revealed significant effects on the development of quorum cheaters (Fig. 1b, c). Furthermore—although the planktonic and biofilm experimental setups are certainly difficult to directly compare—these antibiotic-dependent effects appeared earlier (already at 3 versus only at 6 days) and were more pronounced (a consistent increase of ~50% over the 3–9 days’ time range) in the biofilm than a planktonic mode of growth.

### Impact of low-dose antibiotic concentrations on the development of biofilm- and non-biofilm-associated infection

**Fig. 2:**
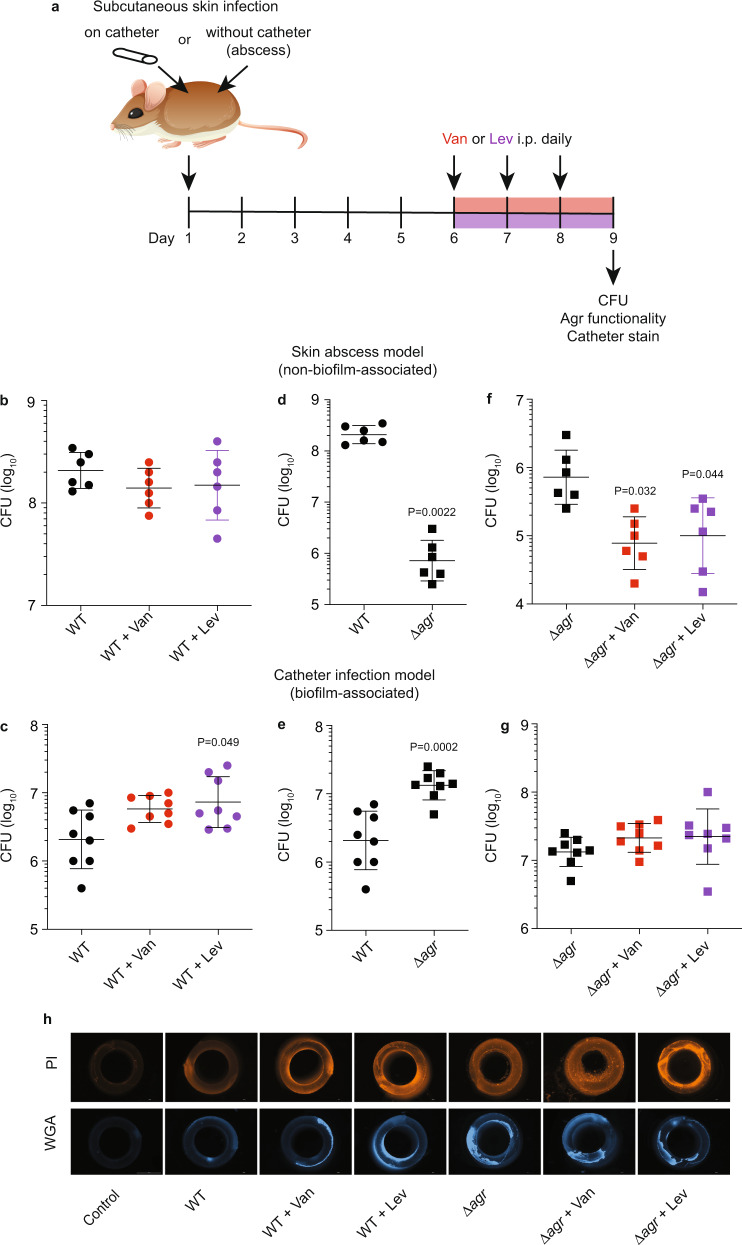
Development of Agr dysfunctionality and impact of antibiotic treatment during device- and non-device-associated infection. **a** Experimental setup. Female, 6–8 weeks old C57BL/6 were used. For antibiotic treatment, mice received antibiotics starting on day 6 after infection (catheter and skin abscess models) every 24 h for 3 days until the end of the experiment. Controls received no antibiotic. Mice received intraperitoneal injections of 0.25 ml containing 0.3 mg ml^−1^ Van (3.75 mg kg^−1^) or oral doses of 0.4 ml containing 0.04 mg ml^−1^ Lev (0.8 mg kg^−1^). All mice in one infection type were infected with the same CFU (skin abscess, ~1 × 10^7^ CFU; catheter infection, 1 × 10^3^–10^4^ CFU with actual adherent bacterial numbers tested.) **b**, **c** Results obtained with infection by *S. aureus* LAC wild-type with and without antibiotics. **d**, **e** Results obtained with infection by *S. aureus* LAC wild-type versus isogenic Δ*agr* mutant. **f**, **g** Results obtained with infection by *S. aureus* LAC Δ*agr* with and without antibiotics. *n* = 6 (**b**, **d**, **f**); *n* = 8 (**c**, **e**, **g**). **h** Representative infected or control (no infection) catheter pieces stained with PI or WGA-Alexa FluorTM 350. Statistical analysis is 1-way ANOVA (**b**, **f**) or Kruskal–Wallis (**c**, **g**), with Dunnett’s post-tests versus data obtained with wild-type control, Mann–Whitney test (**d**), and unpaired, two-tailed *t*-test (**e**). Parametric versus non-parametric tests were chosen based on normal distribution (Shapiro–Wilk) tests for the data in the respective comparisons. Error bars show the geometric mean and geometric SD.

Then, we proceeded to our main goal of analyzing quorum cheating under antibiotic treatment during experimental infection. To that end, we again used strain LAC (USA300) and mouse infection models of biofilm-associated and non-biofilm-associated subcutaneous skin infection (Fig. 2a). Except for device insertion, these two models are very similar and thus offer the possibility to compare for the effect of device (biofilm) involvement. Antibiotic treatment was initiated at day 6 post-infection (in both skin abscess and catheter infection models) and repeated another two times every 24 h, for a total of 3 days, after which CFU was counted. For the investigation of the impact of antibiotics, we focused on Van and Lev and used a low-dose regimen corresponding to ~1/8 of the mouse body weight-corrected dose suggested for the clinical treatment of bone and joint infections^47^. This was intended to mimic the situation frequently encountered during the treatment of device-associated infections, where the applied dosage is subinhibitory due to increased tolerance to antibiotics of the device-associated biofilms, resulting in the persistence of the infection. The low-dose regimen was also necessary because of the comparison to non-catheter-associated skin infection, where higher doses would have rapidly killed all bacteria.

As intended, treatment with Van or Lev only slightly and did not significantly reduce CFU in the skin abscess model (Fig. 2b). Contrastingly, in the biofilm-associated subcutaneous catheter model, CFU was increased by the addition of antibiotics, which was significant for Lev (Fig. 2c). Hypothesizing that the underlying reasons were associated with quorum cheating and its effect on biofilm-mediated resistance, we first determined the behavior of a constructed isogenic QS (Δ*agr*) mutant versus wild-type LAC. In accordance with what we showed previously^32^, the Δ*agr* mutant had strongly and significantly decreased infectivity in the skin abscess model (Fig. 2d), as expected from the control by Agr of several virulence factors that are known to be crucial in that setting, such as PSMs or α-toxin^48,49^. In contrast, the Δ*agr* strain showed significantly higher infectivity than the wild-type strain in the biofilm-associated catheter infection model (Fig. 2e). In the skin abscess model, both Van and Lev treatment led to significantly reduced CFU (Fig. 2f), indicating that in vivo resistance to the used antibiotic concentrations is strongly diminished in the Δ*agr* strain, a likely consequence of the strongly diminished bacterial survival capacities that are associated with Agr dysfunctionality in that setting^32,49^. Notably, the significant increase in CFU in the biofilm-associated catheter infection model that we observed with antibiotic treatment and infection by wild-type *S. aureus* was not observed with the isogenic Δ*agr* strain (Fig. 2g), suggesting that Agr quorum sensing is causally related to this phenomenon. We also stained catheters with PI or WGA-Alexa FluorTM 350, which stain biofilm extracellular DNA or polysaccharide, respectively, confirming that antibiotics increased biofilm formation in the LAC but not Δ*agr*-infected animals (Fig. 2h).

**Fig. 3:**
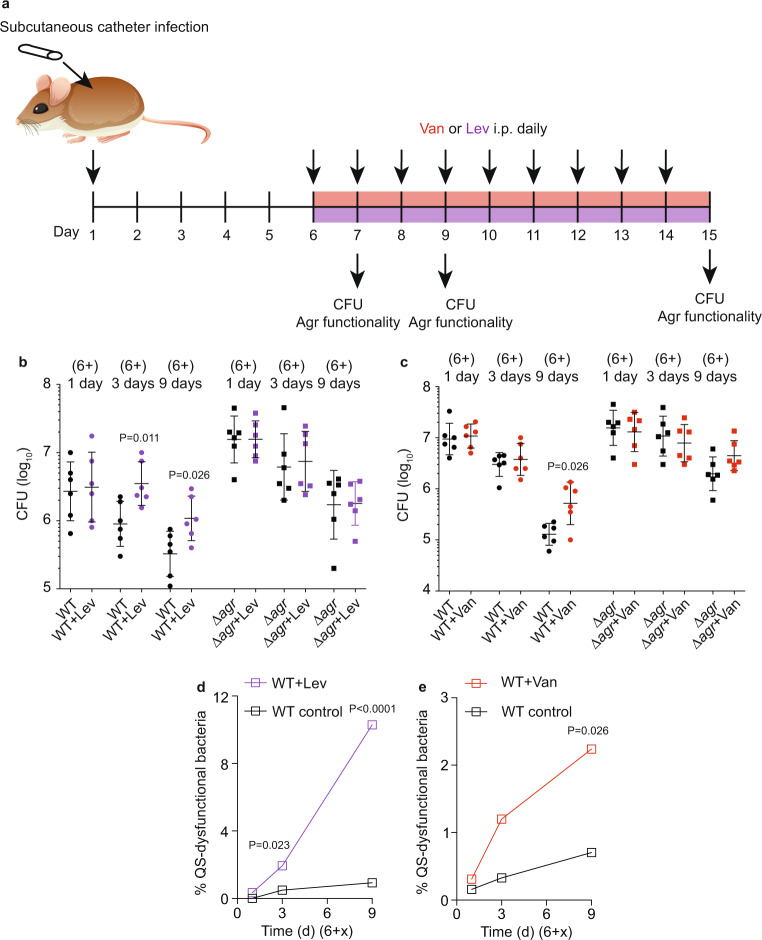
Analysis of the development of Agr (QS)-dysfunctional mutants in the catheter infection model over time. **a** Experimental setup. Mice were implanted with catheters with 1 × 10^3^–10^4^ adherent bacterial CFU and received intraperitoneal injections of 0.25 ml containing 0.3 mg ml^−1^ Van (3.75 mg kg^−1^) or oral doses of 0.4 ml containing 0.04 mg ml^−1^ Lev (0.8 mg kg^−1^) daily for 9 days after infection. Controls received no antibiotic. Different groups of mice (*n* = 6/group) were euthanized on days 1, 3, or 9 after initiation of antibiotic treatment. **b**, **c** CFU on the catheters in the different groups after euthanizing. Statistical analysis is by Mann–Whitney tests. Error bars show the geometric mean and geometric SD. **d**, **e** Agr (QS) dysfunctionality was assessed by hemolysis on sheep agar plates. Statistical analysis is by Fisher’s exact test at every time point based on the underlying raw data (hemolysis phenotype) of analyzed clones.

The short-duration experiment (6 plus 3 days) was chosen to be able to compare directly to non-biofilm-associated skin infection, in which pathogenesis-related phenotypes occur early. However, because effects in the catheter infection model were not very pronounced at 9 (6 plus 3) days post-infection, only reaching significance for Lev in the important comparison shown in Fig. 2c, we performed an additional catheter infection experiment in which we prolonged the infection and took samples at 6 plus 1, 3, or 9 days to obtain time-dependent data (Fig. 3a). The phenomenon of increased infectivity under antibiotic treatment developed over time, reaching significance at 3 and 9 days for Lev and at 9 days for Van, but was completely absent in mice that were infected with Δ*agr* instead of wild-type *S. aureus* (Fig. 3b, c).

**Fig. 4:**
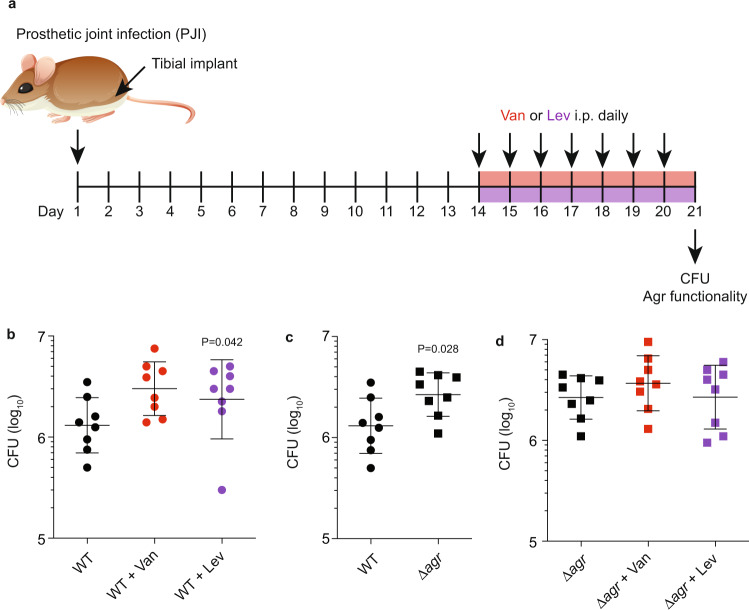
Prosthetic joint infection (PJI) model. **a** Experimental setup. Female, 6–8 weeks old C57BL/6 were used. For antibiotic treatment, mice received antibiotics starting on day 14 after infection every 24 h for 7 days until the end of the experiment. Controls received no antibiotic. Mice received intraperitoneal injections of 0.25 ml containing 0.3 mg ml^−1^ Van (3.75 mg kg^−1^) or oral doses of 0.4 ml containing 0.04 mg ml^−1^ Lev (0.8 mg kg^−1^). All mice were infected with the same CFU (~1 × 10^7^ CFU in 50 μl, intra-articular injection). **b** Results obtained with infection by *S. aureus* LAC wild-type with and without antibiotics. **c** Results obtained with infection by *S. aureus* LAC wild-type versus isogenic Δ*agr* mutant. **d** Results obtained with infection by *S. aureus* LAC Δ*agr* with and without antibiotics. *n* = 8. Statistical analysis is 1-way ANOVA with Dunnett’s post-tests versus data obtained with wild-type control (**b**, **d**) and by unpaired, two-tailed *t*-test (**c**). Error bars show the geometric mean and geometric SD.

Furthermore, we used an additional biofilm-associated infection model, a model of PJI, to corroborate our results obtained in the catheter infection model (Fig. 4a). In this model, we also observed significantly increased CFU in Δ*agr* as compared to LAC-infected animals and increased CFU in LAC but not Δ*agr*-infected animals under antibiotic treatment (reaching statistical significance with Lev) (Fig. 4b–d).

### Development of quorum cheaters during biofilm- and non-biofilm-associated infection with low-dose antibiotic concentrations

If our hypothesis is correct that QS dysfunctionality underlies the observed increased infectivity under antibiotic treatment in the biofilm-associated models, we expect to detect the development of quorum cheaters during infection with wild-type *S. aureus*. To confirm this hypothesis, we determined the hemolytic capacity of isolates as a readout for Agr functionality at the end of the infection. While Agr-dysfunctional mutants were not detected in non-catheter (non-biofilm)-associated skin infection under any condition, they developed in a catheter (biofilm)-associated infection at significantly higher rates (Table 1). Importantly, the rate of Agr-dysfunctional mutant development in catheter-associated infection was further increased significantly under treatment with Lev or Van. Similar observations were made in the PJI model, where no Agr-dysfunctional mutants were observed without, but at significant rates with Lev or Van treatment (Table 1). All non-hemolytic clones interpreted as *agr* mutants were confirmed by DNA sequencing to harbor non-synonymous mutations in the Agr system (Tables 2 and 3). As reported previously for *agr* mutations that occur in vitro or in vivo^32,40,41^, mutations mapped to the *agrA* or *agrC* genes. Interestingly, we frequently detected the same mutation in two or more isolates from one mouse, indicating the proliferation of *agr*-mutated clones. Finally, while the percentage of QS mutants after 6 plus 3 days of intervention was still low, it is important to stress that in our time-extended antibiotic intervention model, the percentage of QS mutants that were detected after 6 plus 9 days was substantial (Lev > 10%; Van > 2% of the entire population) (Fig. 3d, e). The mouse biofilm-associated infection models can barely be further extended because the infections heal. Nevertheless, these data indicate that QS mutants can develop and substantially proliferate during antibiotic treatment of biofilm-associated infection. Because the biofilm phenotypes we observed with low-dose antibiotics can theoretically also be due to reduced Agr expression under those conditions, we analyzed whether low-dose antibiotic treatment leads to changes in *agr* expression. While it is difficult to estimate the actual in vivo concentrations at the biofilm infection site, we used the 5× MIC concentrations we had employed in the in vitro experiment shown in Fig. 1c for that purpose. We believe that the moderate growth inhibition observed with those concentrations in vitro indicates that they are at least as high as those encountered in vivo because we did not observe growth inhibition in vivo with the used antibiotic concentrations at the initial 1-day time points. There was no inhibition of *agr* expression, as measured by qRT-PCR of the *agrA* gene, by 5× MIC of Lev, Van, or Cli (Supplementary Fig. 2). For all three antibiotics, *agr* expression, in fact, rather increased.

**Table 1:** Agr mutation rates during infection under 3-day low-dose antibiotic treatment.

Mutation rate (*agr* mutant clones/total)	Skin abscess	Catheter infection	PJI
Without antibiotic	0 (0/920)	5.88 × 10^−3^ (5/850)	0 (0/914)
Levofloxacin	0 (0/908)	1.75 × 10^−2^ (15/855; *p* = 0.040^a^)	5.5 × 10^−3^ (5/903; *p* = 0.030^a^)
Vancomycin	0 (0/916)	1.74 × 10^−2^ (16/918; *p* = 0.028^a^)	5.6 × 10^−3^ (5/895; *p* = 0.030^a^)

^a^Statistical analysis is by two-tailed Fisher’s exact test versus data obtained without antibiotics in the same infection type.

**Table 2:** Non-synonymous mutations in non-hemolytic clones that developed in the mouse catheter model.

Treatment	Sample no.	Mouse no.	Mutated gene	Mutated location in gene	Amino acid exchange
3d none	1	1	*agrA*	C520A	His174Asn
	2	1	*agrA*	C520A	His174Asn
	3	1	*agrC*	C1160T	Ser387Phe
	4	2	*agrA*	G683A	Cys228Tyr
	5	4	*agrA*	C355T	Arg119Stop
3d Lev	1	1	*agrA*	C520A	His174Asn
	2	1	*agrA*	C520A	His174Asn
	3	1	*agrA*	C520A	His174Asn
	4	1	*agrA*	G683A	Cys228Tyr
	5	1	*agrA*	G683A	Cys228Tyr
	6	2	*agrA*	G683A	Cys228Tyr
	7	2	*agrA*	G683A	Cys228Tyr
	8	2	*agrA*	G541A	Glu181Lys
	9	2	*agrA*	G541A	Glu181Lys
	10	3	*agrC*	C634T	Arg212Cys
	11	3	*agrA*	G443A	Gly148Asp
	12	3	*agrA*	G443A	Gly148Asp
	13	3	*agrA*	G653A	Arg218Gln
	14	3	*agrA*	G653A	Arg218Gln
	15	4	*agrC*	C insertion before T634	Frameshift
3d Van	1	1	*agrA*	C505T	His169Tyr
	2	1	*agrA*	C505T	His169Tyr
	2	1	*agrC*	T1070G	Ile357Asn
	3	1	*agrA*	G227A	Arg76His
	4	2	*agrC*	C1085T	Ser362Leu
	4	2	*agrC*	T557A	Phe186Tyr
	5	2	*agrC*	C1160T	Ser387Phe
	6	2	*agrC*	C1160T	Ser387Phe
	7	2	*agrC*	C1160T	Ser387Phe
	8	2	*agrC*	C1160T	Ser387Phe
	9	3	*agrA*	C520A	His174Asn
	10	3	*agrA*	C520A	His174Asn
	11	3	*agrA*	C520A	His174Asn
	12	3	*agrA*	C520A	His174Asn
	13	4	*agrC*	A796 deletion	Frameshift
	14	4	*agrC*	A796 deletion	Frameshift
	15	4	*agrC*	G1018A	Ala340Thr
	16	4	*agrC*	G1018A	Ala340Thr

**Table 3:** Non-synonymous mutations in non-hemolytic clones that developed in the mouse PJI model.

Treatment	Sample no.	Mouse no.	Mutated gene	Mutated location in gene	Amino acid exchange
3d none	No non-hemolytic clones observed				
3d Lev	1	2	*agrC*	C541T	Gln181Stop
	2	2	*agrA*	G683A	Cys228Tyr
	3	2	*agrC*	C640T	Gln214Stop
	4	2	*agrC*	A1252G	Asn418Asp
	5	3	*agrC*	G1024A	Glu342Lys
3d Van	1	1	*agrA*	C136T	Gln46Stop
	2	2	*agrC*	G940A	Glu314Lys
	3	2	*agrC*	G940A	Glu314Lys
	4	2	*agrC*	G940A	Glu314Lys
	5	3	*agrC*	A898G	Lys300Glu

Altogether, our findings show that antibiotic treatment can exacerbate device-associated *S. aureus* infection and mechanistically link this phenomenon to the increased frequency of development and proliferation of biofilms composed of Agr-dysfunctional mutants that exhibit increased antibiotic resistance.

## Discussion

The exceptional capacity of *S. aureus* and other pathogens to colonize indwelling medical devices to cause persistent, antibiotic-resistant biofilm infections has mainly been attributed to the presence of specific genes, such as those coding for biofilm matrix polysaccharides or proteins^25,50,51^. While such biofilm-associated factors are certain prerequisites for the initiation and persistence of biofilm infection, and the infection environment can also lead to changes in their expression^52^, recent work performed in our laboratories and elsewhere indicates that device-associated infection is a dynamic process with genetic adaptations that play an important role in the progress of such infections. Specifically, mutations in the Agr QS system have now been recognized as defining genetic adaptations developing during and exacerbating *S. aureus* device-associated infection by their increased propensity to form biofilms^31,32,34,40,53,54^, explaining the frequent isolation of such mutants from these infections^34,39,41^.

Because clinical device-associated infections commonly progress under antibiotic treatment, in this study, we aimed to directly investigate whether antibiotic treatment can impact device-associated infections by potentially stimulating quorum cheating, i.e., stimulating the rise of *agr* mutants. In vitro, antibiotics led to earlier and more pronounced development of quorum cheaters in biofilm than in the planktonic mode of growth. More importantly, we show that a low-dose antibiotic regimen exacerbated biofilm-associated infection and was associated with a concomitant rise and proliferation of *agr* mutants.

**Fig. 5:**
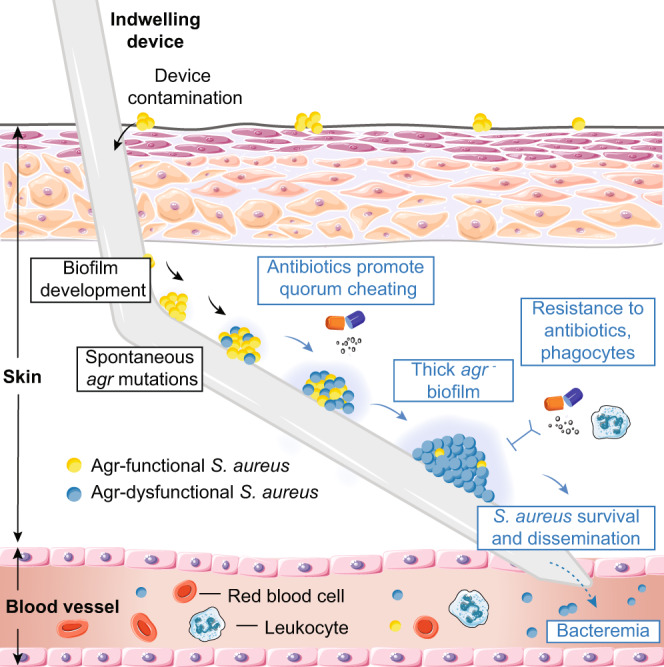
Model of the role of QS (Agr) mutant development during staphylococcal device-associated infection. Naturally, skin-colonizing *S. aureus* contaminates the indwelling medical device (shown as a subcutaneous catheter, as an example) during insertion. Initial colonization of the device by contaminating bacteria ensues. Spontaneous mutations in the *agr* locus happen in some bacteria, producing Agr-dysfunctional *S. aureus*. During prolonged infection under selective pressure by antibiotics (vancomycin, levofloxacin), Agr-dysfunctional mutants outgrow wild-type members of the population. As a result, a strong biofilm forms, considerably increasing antibiotic resistance and resistance to leukocyte attacks. This leads to the exacerbation of device infection, which can involve bacteremia and systemic dissemination.

Our findings are consistent with a model (shown in Fig. 5) in which the persistence of spontaneously arising Agr-dysfunctional QS mutants during biofilm-associated infection is promoted by antibiotic treatment. As our previous investigations have shown^32,40^, biofilms composed of *agr* mutants have increased resistance to antibiotics and leukocyte attacks; thus, the development of Agr-dysfunctional biofilms during clinical biofilm-associated infection is likely to be explained by the selective pressure exerted by a combination of these two mechanisms.

These findings have important clinical implications. The most striking message from our study is that antibiotic treatment at concentrations that do not eradicate the infection—which is the very problem encountered in the case of device-associated infections—can trigger the rise of Agr-dysfunctional quorum cheater mutants and thereby further exacerbate the infection.

Our study has limitations. We only used selected antibiotics for our study. However, we believe that our results can be generalized because (i) the antibiotics we chose cover those frequently used in the clinic, (ii) represent entirely different modes of action (DNA synthesis, cell wall synthesis), and (iii) antibiotic tolerance by biofilm formation is generally recognized to be non-specific. Furthermore, due to limits of how long mouse biofilm-associated experimental infection can be maintained, we were not able to prolong infection until the complete overtake of quorum cheaters that we saw in chronic clinical biofilm infection^40^ could be observed. However, given the considerable expansion of quorum cheaters that we observed in the mouse model during 2 weeks of infection, we believe it is reasonable to assume further spread to the point observed in long-term human infection. In addition, the strong increase under antibiotic treatment, which represents the main message of this study, was clearly visible during the monitored timeframe. Finally, we acknowledge that there may be additional mechanisms unrelated to biofilm formation that facilitate survival of Agr-dysfunctional mutants in persistent infection, such as intracellular survival in host cells, which has been linked to PSMs^55^. While intracellular persistence was not addressed in our study, it has been linked to factors other than Agr, such as small colony variants or the regulator Rsp^56,57^, mutations associated with which were not identified in our previous human study that monitored mutations arising during biofilm infection^40^.

## Methods

### Bacterial strains and growth conditions

*S. aureus* LAC (pulsed-field type USA300) was obtained from NARSA (Network on Antimicrobial Resistance in *S. aureus*). The isogenic *agr* mutant of *S. aureus* USA300 LAC has been described previously^48^. The entire *agr* system is deleted in this mutant, which has been produced via phage transduction from strain RN6911^58^. All strains were stored in tryptic soy broth (TSB and BD) supplemented with 30% glycerol at −80 °C. For pre-cultures, which were used to inoculate main cultures in the various experiments as indicated, bacteria were grown on solid agar plates containing TSB for ~24 h, from which single clones were used for inoculation. Planktonic cultures were grown under shaking conditions (200 rpm) in TSB, and biofilm cultures in microtiter plates in TSBg (TSB plus 0.5% glucose) without shaking. All bacterial cultures were grown at 37 °C.

### Analysis of QS dysfunctionality and agr sequencing

Similarly to several previous studies^32,39,41,45^, we used hemolytic activity upon plating on sheep blood agar as a readout for Agr functionality. Most mutants that lose hemolytic capacity reportedly show spontaneous mutations in the Agr system^32,39,41,45^. In key in vivo experiments, we sequenced the *agrA* and *agrC* genes of the non-hemolytic clones with primers agrACforward (5′-GCTATACAGTGCATTTGCTAG-3′) and agrACreverse (5′-TCGCAGCTTATAGTACTTGTG-3′).

### In-vitro development of Agr-dysfunctional mutants

For the planktonic mode of growth, cultures were grown in TSB for 24 h in 15 ml round-bottom tubes, and fresh cultures were inoculated every day for 9 days using 1/200 volume of the previous culture in only TSB (control) or TSB with levofloxacin (Lev), vancomycin (Van), or clindamycin (Cli) at ¼ MIC. The MICs determined for strain LAC against antibiotics used in this study were: 1 μg/ml (Van), 4 μg/ml (Lev), and 0.3 μg/ml (Cli). On days 3, 6, and 9, dilutions were plated on sheep blood agar, and hemolysis was evaluated after overnight incubation at 37 °C. For the biofilm mode of growth, cultures of *S. aureus* LAC were inoculated at 1/200 volume from pre-cultures into microtiter plate wells containing 200 μl of TSBg and grown for 48 h to form a biofilm. Then, the supernatant was gently removed, and 200 μl TSBg containing Lev, Van, or Cli was dispensed into the wells at 5× MIC. In the following 9 days, every 3 days, the old supernatant was gently removed and replaced with fresh TSBg (200 μl) with the respective antibiotics.

### Antimicrobial resistance profiles

The antimicrobial susceptibilities of isolates were determined by the disc diffusion method on Mueller–Hinton agar according to Clinical and Laboratory Standards Institute (CLSI) guidelines. *S. aureus* ATCC29213 was used as a quality control.

### Quantitative real-time reverse transcription polymerase chain reaction (qRT-PCR)

The total RNA of *S. aureus* was extracted using an RNA extraction reagent (Takara RR047A), then complementary DNA was synthesized from the total RNA by using the RT reagent Kit (Takara, RR037A). Amplification of the resulting complementary DNA sample was performed with the TB Green^®^ Fast qPCR Mix (Takara, RR430A). The 7500 Sequence Detector (Applied Biosystems) was used to perform reactions in MicroAmp Optical 96-well reaction plates. Oligonucleotides used were as follows (5′–3′): gyrB-F, CAAATGATCACAGCATTTGGTACAG, gyrB-R, CGGCATCAGTCATAATGACGAT, agrA-F, GCACATACACGCTTACAATTATTA, agr-R, ACACTGAATTACTGCCACGTTTTAA.

### Animal experiments

Female C57BL/6 mice were purchased from JSJ Corp. (originally introduced from Jackson Laboratory) and used for all mouse experiments. All mice were between 6 and 8 weeks of age at the time of use. All animals were euthanized by CO_2_ at the end of the studies. In the antibiotic treatment experiments, mice received antibiotics every 24 h starting on day 6 after infection (catheter and skin infection models) or day 14 after infection (PJI) every 24 h until the end of the experiment. Controls received no antibiotic. Van and Lev were dissolved in water to a concentration of 0.3 mg ml^−1^ or 0.04 mg ml^−1^, respectively, and solutions were filter-sterilized. Mice received intraperitoneal injections of 0.25 ml containing 0.3 mg ml^−1^ Van (3.75 mg kg^−1^) or oral doses of 0.4 ml containing 0.04 mg ml^−1^ Lev (0.8 mg kg^−1^). Investigators were blinded to group allocation when determining CFU.

Skin abscess model. About 1 × 10^7^ CFU of bacteria in 50 μl of PBS were injected subcutaneously into the left and/or right flank of shaved mice. To determine the bacterial load, abscesses on day 9 were surgically removed, cut into small pieces and split into four separate tubes containing 500 mg of borosilicate glass beads and 500 μl of sterile PBS. The skin pieces were homogenized in a FastPrep 96 (MP Bio) homogenizer, 5 × 1 min at 1800 rpm. The homogenates were then plated, incubated at 37 °C for 24 h, and enumerated.

Catheter infection model. The catheter infection model was performed essentially as described previously^32^. Briefly, 1-cm catheter pieces (Surflo^®^ Teflon i.v. catheters, 14 ×2”) were coated with equal numbers of bacteria. Adherence was tested in control experiments and was in the same range ( ~ 1 × 10^3^–10^4^). Catheters were then inserted under the dorsal skin of mice. Catheters were removed carefully from the skin on days 7, 9, and 15 after infection, and CFU on the catheters and the directly adjacent tissue were determined by plating as above after performing a validated sonication protocol to disrupt bacterial agglomerates on the catheters and homogenizing the tissue samples using the FastPrep 96 (MP Bio) homogenizer, 5 × 1 min. The sonication protocol consisted of sonication of harvested catheters for 5 min in 1 ml PBS in 2 ml tubes in an Ultra Sonic Cleaner, followed by vortexing for 15 min on a Vortex-Genie2. Some catheters obtained on day 9 after infection were also fixed with 2.5% glutaraldehyde in 0.1 M PBS for 30 min and then stained either with 10 μM propidium iodide (Yeasen, Shanghai) (staining extracellular DNA) for 15 min or 2.5 μg/ml wheat germ agglutinin (WGA)-Alexa FluorTM 350 (Invitrogen, W11263) (staining staphylococcal biofilm exopolysaccharide) for 20 min. Stained catheters were then washed with PBS and longitudinally placed in wells of a 96-well sterile, tissue culture-treated microplate with black well walls and an optically clear cyclic olefin bottom and imaged using an Agilent BioTek Lionheart FX Automated Microscope using the confocal channel (555 nm laser for the PI-stained staphylococcal biofilm and 350 nm laser for the WGA-stained staphylococcal biofilm) with the 4× objective.

PJI model. For the PJI model, mice underwent unilateral proximal tibial implant insertion with a 10-mm syringe needle from a 1-ml BD insulin syringe (29 G × 1/2”, 0.33 mm × 12.7 mm) using a previously described surgical technique^59^. Following press-fit implant insertion and arthrotomy closure, a gas-tight syringe (65 RN, Hamilton) was utilized to administer a 50-μl intra-articular injection of bacteria with ~ 1 × 10^7^ CFU. Beginning CFU dosing was verified through parallel syringe injections placed directly in triplicate onto sheep blood agar plates. On day 21 after infection, mice were euthanized by CO_2_. Each of the whole unilateral tibial implant with the syringe needle and the directly surrounding tissue was removed and CFU were determined by plating as above, after performing a validated sonication protocol (see above) to disrupt bacterial agglomerates on the total unilateral tibial implant, and homogenizing the tissue samples using the FastPrep 96 (MP Bio) homogenizer, 5 × 1 min.

### Statistics

Statistical analysis was performed using GraphPad Prism version 9.3.1 for Mac OS. Unpaired, two-tailed *t*-tests or Mann–Whitney tests were used for the comparison of two groups depending on the results of Shapiro–Wild tests for normality of distribution, and 1-way or 2-way ANOVAs, or Kruskal–Wallis tests, as applicable and depending on Shapiro-Wilk tests, for the comparison of more than two groups. The specific tests used are indicated in the figure legends. For arithmetic scales, error bars depict the standard deviation (SD) and lines depict the mean. For logarithmic scales, the geometric mean with the geometric SD is depicted. All replicates are independent.

### Graphics software and pictures

Illustrations were made using Biorender and Adobe Illustrator under NIAID licenses. Mouse pictures are from Vecteezy.com.

### Inclusion and ethics

All animal work complied with all relevant ethical regulations and was approved by the Ethics Committee of Ren Ji Hospital, School of Medicine, Shanghai Jiao Tong University, Shanghai, China (Approval Number: KY2021-225-B).

### Reporting summary

Further information on research design is available in the Nature Research Reporting Summary linked to this article.

## Supplementary information

Supplementary Material

Reporting Summary

## Data Availability

All data generated or analyzed during this study are included in this published article or in the supplementary information files. The *S. aureus* LACΔ*agr* strain is available from Dr. Min Li or Dr. Michael Otto subject to a simple transfer agreement.
