# Supplementary material for: Antibiotic treatment can exacerbate biofilm-associated infection by promoting quorum cheater development
Source: NPJ Biofilms Microbiomes. 2023 May 18;9:26. doi: 10.1038/s41522-023-00394-4 (PMC10195787; doi:10.1038/s41522-023-00394-4)
Supplement: Supplementary file 1 — Supplementary Material [file 41522_2023_394_MOESM1_ESM.pdf]

Antibiotic treatment can exacerbate biofilm-associated infection by promoting quorum cheater development

Lei He, Huiying Lv, Yanan Wang, Feng Jiang, Qian Liu, Feiyang Zhang, Hua Wang, Hao Shen, Michael Otto, Min Li

Supplementary Material

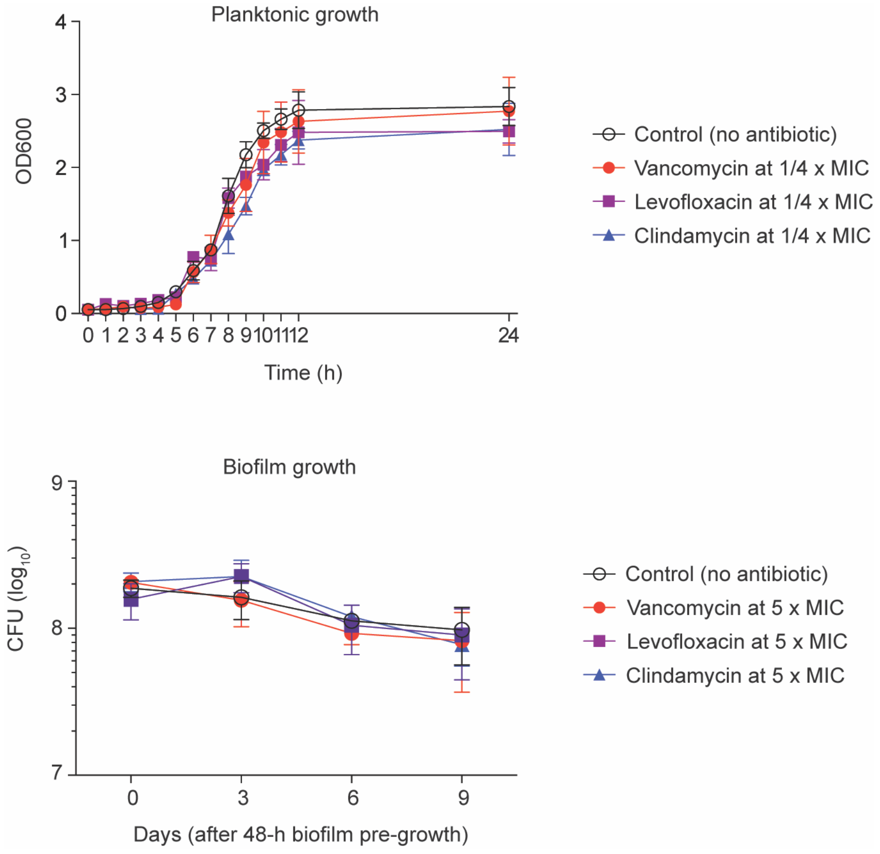

Supplementary Figure 1. Growth curves in planktonic and biofilm modes of growth of the used *S. aureus* strain LAC with and without the applied sub-inhibitory concentrations of antibiotics at ¼ × MIC and 5 × MIC, respectively.

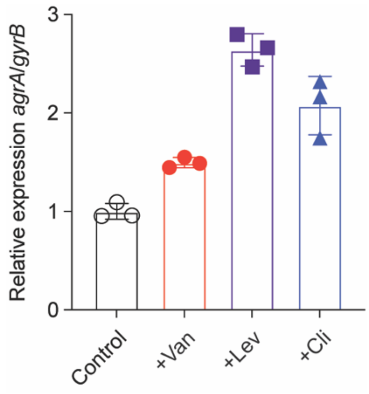

Supplementary Figure 2. Agr expression with low-dose antibiotics. Expression of *agr* in biofilm mode was determined by qRT-PCR of *agrA* with antibiotics used at 5 × MIC.
